# Artificial tektites: an experimental technique for capturing the shapes of spinning drops

**DOI:** 10.1038/srep07660

**Published:** 2015-01-07

**Authors:** Kyle A. Baldwin, Samuel L. Butler, Richard J. A. Hill

**Affiliations:** 1 School of Physics and Astronomy, University of Nottingham, Nottingham, NG7 2RD, UK; 2 Department of Geological Sciences, University of Saskatchewan, Saskatoon Saskatchewan, S7N 5E2, Canada

## Abstract

Determining the shapes of a rotating liquid droplet bound by surface tension is an archetypal problem in the study of the equilibrium shapes of a spinning and charged droplet, a problem that unites models of the stability of the atomic nucleus with the shapes of astronomical-scale, gravitationally-bound masses. The shapes of highly deformed droplets and their stability must be calculated numerically. Although the accuracy of such models has increased with the use of progressively more sophisticated computational techniques and increases in computing power, direct experimental verification is still lacking. Here we present an experimental technique for making wax models of these shapes using diamagnetic levitation. The wax models resemble splash-form tektites, glassy stones formed from molten rock ejected from asteroid impacts. Many tektites have elongated or ‘dumb-bell' shapes due to their rotation mid-flight before solidification, just as we observe here. Measurements of the dimensions of our wax ‘artificial tektites' show good agreement with equilibrium shapes calculated by our numerical model, and with previous models. These wax models provide the first direct experimental validation for numerical models of the equilibrium shapes of spinning droplets, of importance to fundamental physics and also to studies of tektite formation.

The question of how a spinning liquid drop deforms under the action of rotation can be traced back as far as the debate between Newton and Cassini concerning the shape of the Earth^1^. While Cassini favoured an Earth elongated at the poles, Newton correctly calculated the opposite: that the competing effects of self-gravitation and centrifugal force would deform the Earth, or indeed any self-gravitating and rotating body, into an oblate shape, flattened at the poles and expanded at the equator. Later, Joseph Plateau, realizing that the cohesive effects of surface tension in a liquid drop act similarly to self-gravitation in holding a large mass together, set out to observe the shape of a spinning droplet in a neutral-buoyancy experiment^2^. With increasing angular momentum the oblate-like shapes observed by Plateau eventually became unstable to an ellipsoid-like shape, which, with increasing angular momentum, evolved into a two-lobed ‘dumb-bell' shape. The *equilibrium* shapes of a rotating droplet are of particular interest: the problem of a rotating droplet held together by surface tension is a special case of the more general problem of the charged rotating droplet, which unites the theory of the shapes of classical charged droplets, of importance to nuclear physics^3^,^4^,^5^,^6^, with the theory of the shapes of self-gravitating masses^7^,^8^,^9^,^10^,^11^. Chandrasekhar was able to find an analytic solution for the shape of *axisymmetric* spinning droplets held together by surface tension^12^,^13^, but there are no analytical solutions for the shapes of more rapidly rotating *non-axisymmetric* droplets, which must be obtained numerically. Developments in computing have enabled increasingly sophisticated numerical calculations of the shapes of equilibrium^14^,^15^ and non-equilibrium^16^ spinning droplets. Here, we present experimental measurements that can be used to validate such models: we determine the dimensions of the stable equilibrium shapes experimentally and compare the results of our experiments with our numerical model and with earlier calculations. Our experimental approach uses diamagnetic levitation to manufacture ‘artificial tektites' from spinning molten wax droplets. Details of the technique of diamagnetic levitation, including its stability, have been published elsewhere^17^,^18^,^19^,^20^,^21^,^22^,^23^,^24^,^25^,^26^,^27^,^28^. We levitated between 0.1 and 0.5 ml drops of liquid wax and spun the drop with air flow from two air nozzles directed tangential to the surface, as shown in Fig. 1. The magnetic force levitating the liquid acts throughout the body of the liquid and does not distort the shape of the droplet, avoiding a common problem with other levitation techniques; our measurements show that the levitating liquid droplet is close to spherical at rest. By rapidly cooling the molten levitating droplet, inducing solidification, we freeze the shape of the droplet for later examination. Since Plateau's technique of suspending the droplet in an immiscible liquid suffers from the effects of viscous drag from the surrounding liquid, subsequent experiments to observe the shapes of spinning droplets have been performed using droplets in air, either on orbiting spacecraft^29^, by rolling droplets down an inclined plane^30^,^31^, or by using acoustic^32^ and magnetic levitation^23^,^24^. These studies typically track the evolution of the shape of the droplet by measuring the length of the longest axis of the droplet and/or identifying bifurcations in the shape as a function of angular velocity and angular momentum. Accurate three dimensional measurements of the droplet's shape have, until now, not been obtained owing to the technical challenge of observing simultaneously all three symmetry axes of the droplet during rotation. Here, solidifying the liquid droplet allows us to make direct measurements of the dimensions of the droplet, once removed from the magnet.

## Results

The still images in Fig. 2 show a time lapse sequence of a wax drop, from pipetting the liquid wax into the magnetic field to complete solidification. In this experimental run, the droplet was spun up until it developed a two-lobed shape. In these images, the camera was pointing down the magnet bore, with the optical axis aligned with the rotation axis of the droplet, which coincides with the axis of the cylindrical bore. The sequence starts at *t* = 0 s with an image showing the withdrawal of the pipette after formation of the droplet. The air flow was activated at *t* = 20 s. As the angular velocity of the droplet increased, its equatorial radius expanded, initially retaining an axisymmetric shape, as can be seen in the fourth (*t* = 30 s) and fifth (*t* = 40 s) images. This shape eventually became unstable to a tri-axial shape (*t* = 50 s), which with increasing angular momentum quickly evolved into a two-lobed shape (*t* = 60 s). At this point in this experimental run, the air flow was reduced to a level sufficient to maintain constant angular velocity, allowing the droplet to approach rigid body rotation. The droplet continued to rotate as a rigid body with no discernible change in the longest symmetry axis for ~20 s. At *t* = 80 s solidification began at the periphery of the two lobes, with complete surface solidification after a further 20 s. The total time from pipetting to complete surface solidification was of order 100 seconds, comparable to the estimated cooling time for some natural tektites^33^,^34^.

Numerous ‘artificial tektites' were produced by this method, with a range of angular velocities and droplet volumes between 0.1 and 0.5 ml. Fig. 3 a) shows a sample of the shapes formed over the course of these experiments. Fig. 3 b) shows a set of artificial tektites that have volume of *V* = 0.5 ml. Each of these wax models were formed from liquid droplets that were spinning with different angular momenta when they solidified. From top to bottom, as pictured in Fig. 3 b), the wax shapes solidified from drops rotating with increasing angular momentum. In this set, all the shapes possess reflection symmetry about three orthogonal axes passing through the centre of mass of the shape. With increasing angular momentum the shapes progress from a sphere (not rotating) to oblate-like to tri-axial (three unequal symmetry axes), and finally two-lobed ‘dumb-bells'. In the oblate-like and tri-axial shapes, the shortest symmetry axis coincides with the axis of rotation. In ‘dumb-bell' shapes the axis of rotation is perpendicular to the line joining the centres of the two lobes. We were not able to observe a more elongated droplet than the one at the bottom of the figure because we observed that the droplet fissioned at the central neck between the lobes.

**Figure 1:**
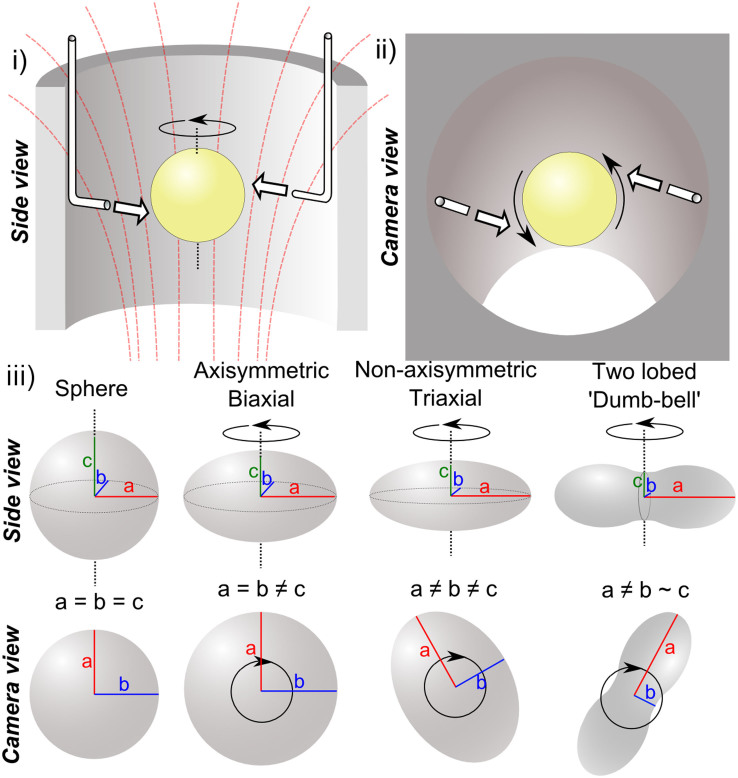
Schematic of a spinning levitating drop inside the magnet bore as viewed from i) the side, ii) above (camera view). Direction of air flow is indicated by white arrows. Solid black arrows show the direction of rotation. Magnetic field lines are drawn in red. iii) Evolution of the droplet shape (schematic) with increasing angular velocity. The three symmetry axes are labelled *a*, *b* and *c*, where *a* is the longest axis, *c* coincides with the axis of rotation, and b is perpendicular to *a* and *c*.

**Figure 2:**
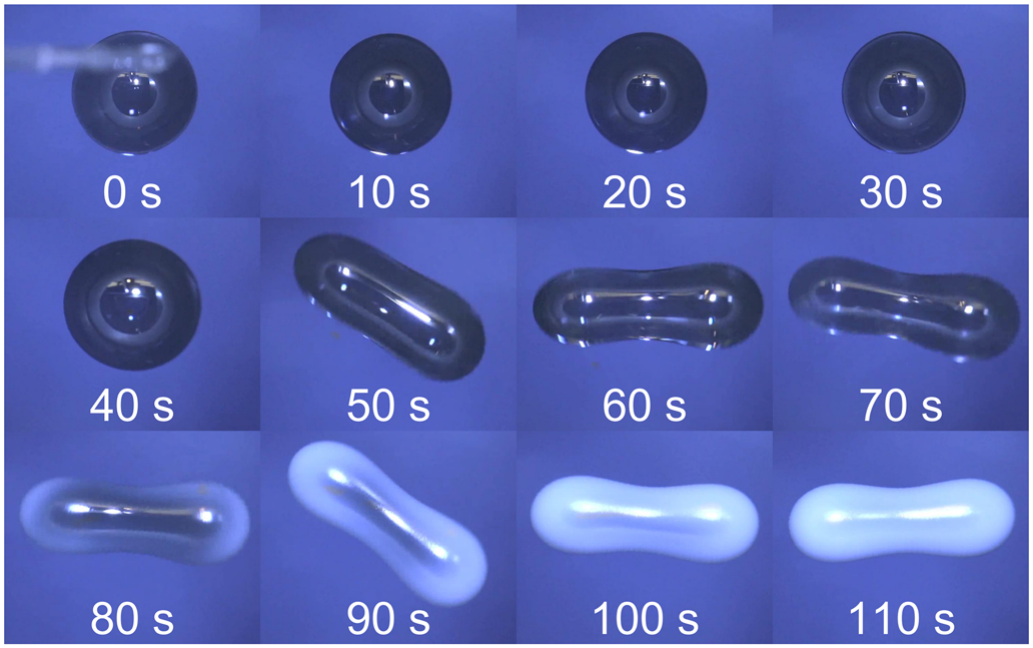
Time lapse sequence of images of the solidification of a wax droplet (*V* = 0.4 ml) viewed from above, from pipetting (top left) to completely solidified (bottom right).

**Figure 3:**
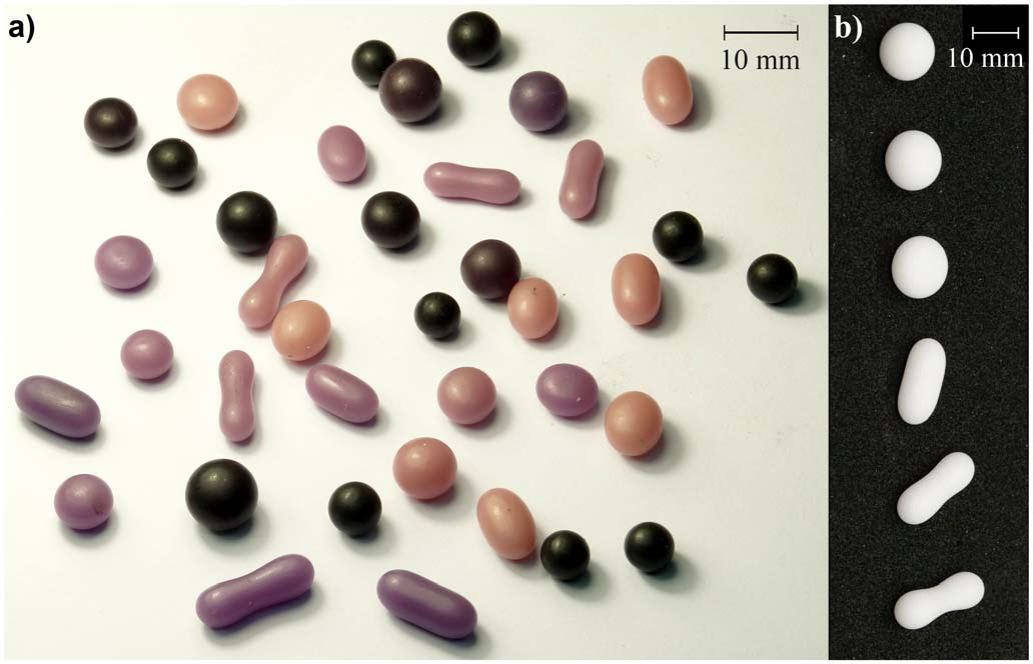
(a) Assortment of wax models. (b) The shapes of solidified wax droplets (0.5 ml) with increasing angular momentum from top to bottom.

**Figure 4:**
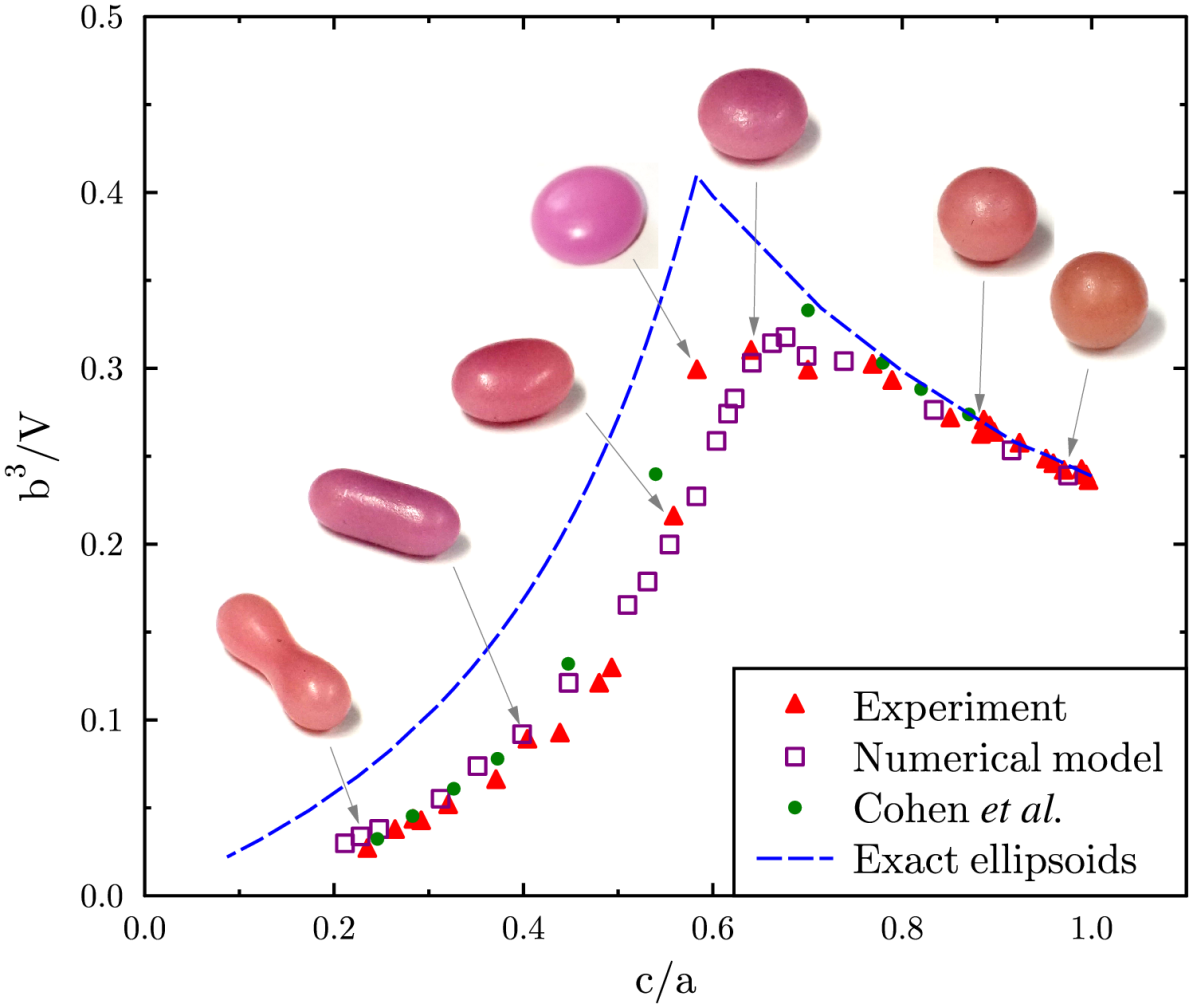
Ratios of the lengths of the major axes, *a*, *b* and *c*. Here *V* is the droplet volume. Triangles: experimental data. Squares: results from the numerical model. Circle: point of bifurcation to a two lobed dumb-bell shape. Filled circles: ratios reported by Cohen *et al*.^7^. Dashed line: exact ellipsoids (oblate for *c*/*a* > 0.58, prolate for *c*/*a* < 0.58).

Fig. 4 shows the dimensionless ratios of the lengths of the symmetry axes of the tektites, plotted as *b*^3^/*V* versus *c*/*a*, where 2*a* is the length of the longest axis, 2*c* is the length of the axis that coincides with the axis of rotation, and 2*b* is the length of the axis perpendicular to a and *c*, as illustrated in Fig. 1. Data from experiment and data from the numerical model are plotted. Each experimental data point corresponds to an individual wax tektite model. The data point at *c*/*a* = 10 represents the axial ratios of a sphere. Data points at progressively smaller *c*/*a* values represent droplets with progressively larger angular momenta. Also plotted are ratios reported by Cohen *et al*.^7^ for uncharged, surface tension-bound droplets. The corresponding axial ratios of the equilibrium shapes of gravitationally bound and spinning masses (exact ellipsoids), are shown by the blue dashed line.

## Discussion

Our initial calculations of the stable equilibrium shapes showed a poor fit with experiment for the most strongly deformed prolate shapes. Subsequently, the numerical calculation was stopped part way through and the mesh was recalculated on the deformed geometry. When this was done, the results of our numerical calculations show good agreement for all values of *c*/*a*, as shown in the figure. Our results are also in reasonable agreement with earlier calculations compiled by Cohen *et al.*^7^.

The change in the gradient of the graph at *c*/*a* ≈ 0.67 marks the switch in stability from the axisymmetric equilibrium shapes to tri-axial equilibrium shapes with increasing angular momentum^7^,^14^. Relatively few wax shapes were produced with 0.5 < *c*/*a* < 0.65 because, during spin-up with constant airflow, the torque on the droplet changes rapidly immediately after axisymmetry has been broken, as the arms of the droplet catch the airflow like the sails of a windmill. This effect made it difficult to maintain a constant angular velocity in this region of *c*/*a*, especially since simulations show that this range of *c*/*a* corresponds to a very narrow window of angular momentum.

Data points with *c*/*a* ≤ 0.45 correspond to tektites that have sufficient angular momentum to form a two-lobed shape with an identifiable neck. For these shapes, the dimensions *b* and *c* are the dimensions of the neck, not the lobes, as illustrated in Fig. 1. We did not observe stable dumb-bells experimentally with *c*/*a* ≤ 0.22, or with simulation for *c*/*a* < 0.2 owing to the loss of equilibrium at the corresponding angular momentum; experimentally, we observed an accelerating narrowing and rapid pinch-off of the central neck between the two lobes at this point, leading to fission of the two lobes, often accompanied by the production of one or more small satellite droplets between the lobes (see also *e.g.*^29^,^35^).

The shapes of gravitationally bound objects are subtly different from ones held together by surface tension alone^7^. Consistent with this, both experimentally-measured and numerically calculated axial ratios differ from that expected of gravitationally-bound equilibrium shapes. For gravitationally-bound droplets, the equilibrium shape of the droplet progresses through a sequence of exact ellipsoids with increasing angular momentum, beginning from spherical at rest^12^. At low angular momentum the stable equilibrium shape is an oblate (Maclaurin) spheroid^12^. The oblate spheroids become secularly unstable to tri-axial (Jacobi) ellipsoids above a critical angular momentum^7^,^36^. Both experiment and simulation are clearly capable of resolving the subtle difference between the shapes of the stable equilibrium shapes of surface-tension bound droplets and the exact ellipsoids of Jacobi beyond the bifurcation point. In the region of oblate-like shapes (two symmetry axes equal), a small difference is discernible between our results (experimental and numerical) and the Maclaurin shape series, up to *c*/*a* ≈ 0.75. Beyond this, the two shapes cannot be distinguished clearly within experimental uncertainty.

To obtain these experimental data, we kept the droplet spinning at constant angular velocity for a minimum of 10 seconds prior to solidification in order to allow the droplet to reach a state of solid body rotation. In addition, we also observed droplets that solidified during spin-up. These formed non-equilibrium shapes suggestive of some natural tektite forms observed in the field^30^,^37^.

Although it is possible to levitate wax droplets with volume *V* > 0.5 ml, we observed wrinkling and dimpling in the solidifying surface layer of such drops prior to complete surface solidification. Solidification is non-uniform owing to the cooling air flow impacting the drop primarily in the azimuthal plane. This non-uniform solidification acts to deform the shape of larger droplets as the advancing solid-liquid boundary leaves behind a soft solid surface that continues to contract upon further cooling, resulting in dimples and ridges in the final solid structure, distorting the final shape of the droplet. For this reason we limited our analysis to drops ≤ 0.5 ml. These features may be analogous to some surface features such as schlieren seen in natural tektites^37^.

Stable diamagnetic levitation requires a restoring force to maintain the droplet levitating in stable mechanical equilibrium. This restoring force can be treated as an effective gravitational acceleration, g*, acting toward the equilibrium levitation point, which acts to deform the shape of the drop toward the shape of the trap^38^. In our experiments, g* ~ 0.1 ms^−2^. For wax, with a surface tension *γ* ≈ 30 mJ m^−2^ and density *ρ* ≈ 0.9 g cm^−3^, the capillary length is thus 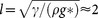 cm. This value is about twice the largest diameter of the droplets considered here, indicating that the restoring force has a small influence on the shape of the droplet compared to surface tension. The good agreement between our experimental and numerical data supports this view, given that the numerical simulation does not include the restoring forces involved in levitation.

## Conclusion

With the use of diamagnetic levitation, we have developed a technique for observing, and capturing, the stable equilibrium shapes of spinning liquid drops of up to 0.5 ml in volume. This method allowed us to obtain accurate experimental measurements of the dimensions of the stable equilibrium shapes. The results of our numerical model agree well with our experimental data, and are consistent with earlier estimates.

Cohen *et al.* surveyed the approximations that existed in 1974, for the equilibrium shapes of droplets^7^. Our measurements of the shapes of the wax models compare well with estimates found in this paper of the lengths of the symmetry axes of surface-tension bound droplets rotating in stable equilibrium, although our data suggests that, for a particular ratio of *c*/*a*, the *b*-axis of the equilibrium shape of the droplet is slightly smaller than that reported by Cohen *et al.*^7^ in the region close to the bifurcation point. Brown and Scriven first applied finite element methods to calculating these shapes in order to obtain more accurate results^14^, and since then increases in computing power have enabled increasingly sophisticated numerical models. We have compared our experimental data with data from a numerical model that was recently applied to solving the shapes of tektites^16^ and found good agreement between the two, after some fine adjustment was made to the simulation parameters. The agreement between the experiments and the numerical model regarding equilibrium shapes gives us good confidence in both methodologies. We suggest that these results can therefore be used as a benchmark against which to test existing and future models.

In addition to fundamental physics, the problem of stable equilibrium shapes of spinning droplets also arises in geophysics concerning the formation of splash-form tektites^16^,^24^,^37^. Such tektites represent material from the near-surface of Earth's crust that was ejected as a liquid by a large impactor^39^,^40^,^41^. Splash form tektites are found in shapes such as oblate ellipsoids and two-lobed ‘dumbbells' with sizes ranging from ~1 mm to ~10 cm; their shapes are governed mainly by the competing influences of surface tension and rotation^16^, just as the wax droplets in this study are. Teardrop shapes, also common^30^, are thought to result from fission at the neck of the two-lobed shape under rotation. In order to take on forms of revolution, it has been shown that splash-form tektites must have had relatively low relative velocities through the atmosphere (<10 m/s) and rotation rates of order 1 rev/s^30^,^34^, similar conditions to those under which the wax shapes were produced. Since droplets often solidify before reaching a stable equilibrium configuration, factors including rate of cooling^42^,^43^, viscoelasticity^44^, and thermal expansion^45^ also play a role in determining their shape. While many ablate during flight, or are broken or deformed on impact with the ground, some retain their in-flight shapes after impact^37^,^46^; the shapes of such stones can be compared with numerical models to shed light on the processes leading to their formation. Although the focus of the present work was to study the equilibrium shapes of droplets, we also experimented with producing non-equilibrium shapes by solidifying the wax before equilibrium was reached. This suggests that the methodology presented here could be developed in future work to study experimentally the influence of factors thought to influence the shapes of splash-form tektites, (including the teardrop-shaped pieces resulting from fission) such as the viscoelasticity of the molten rock^44^, its rate of cooling during flight^42^,^43^, its thermal history^45^ and spin-down rate^37^. An improved understanding of the effects of ongoing cooling of splash-form tektites on their final shape, coupled with field observations, may help to constrain details regarding the environment in which real tektites formed.

## Methods

### Numerical Model

The numerical model generates the stable equilibrium shapes of a spinning droplet by solving the Navier-Stokes equations with constant fluid density and viscosity in a rotating reference frame. At each time step, the moment of inertia of the drop is calculated and the rotation rate of the reference frame is adjusted so as to conserve the angular momentum of the drop. The centrifugal, Coriolis and Euler or Pointcaré (the force arising from the time rate of change of the angular rotation rate of the reference frame) forces are taken into account. The surface tension on the outer surface of the drop is assumed constant. Surface tension imparts a normal stress on the outer boundary of the drop that is proportional to the curvature. The equations were solved numerically using the finite-element modeling package Comsol Multiphysics which includes an arbitrary Langrange-Eulerian formulation for deforming meshes. The outer boundary of the mesh was required to move at the fluid velocity. The surface tension induced normal stress was included using the formulation described in Walkley *et al*.^47^. The simulations used 80 tetrahedral elements and Winslow smoothing was used to specify the locations of internal elements. The model was started from a nearly unit spherical state: an ellipsoid with one horizontal axis of length 1.01, vertical axis 1 and the other horizontal axis was chosen so that the volume of the sphere was 4*π*/3. The effects of viscosity were parameterized through the Ohnesorge number which was taken to be 1 for all of the simulations here. The equilibrium shape of a liquid drop does not depend on the Ohnesorge number which influences how long the deformation process will take, and only weakly affects the transient shapes the drop evolves through as it approaches equilibrium. The model is described in more detail in Butler *et al*.^16^. Errors appear in the numerical method for the most strongly deformed shapes. For this reason, the numerical simulation was stopped part way through and the mesh was recalculated on the deformed geometry for highly elongated ‘dumb-bell' shaped drops. These remeshed simulations are in better agreement with the angular momentum-angular velocity curves of Brown and Scriven^14^.

### Experimental Technique

We used an 18.5 T superconducting solenoid magnet with a vertical, 58 mm diameter bore (Cryogenic Ltd., London) to levitate the droplets of wax. The temperature of the air in the bore was approximately 18°C. Prior to levitation, the wax was heated above its melting point (which is in the range 54–58°C) using a hot plate, to a temperature of 180 ± 4°C. The liquid wax was pipetted into the magnet bore at the point in the magnetic field where the wax levitated in stable mechanical equilibrium. Previously, Takahashi *et al*. levitated non-rotating wax cubes to obtain precise measurements of magnetic susceptibility^26^. We allowed ~10 s for any center of mass motion, imparted during pipetting, to decay, before the droplet was spun up to the desired angular velocity using air flow from two air nozzles directed along a tangent to the surface, as shown in Fig. 1. Previously, similar apparatus has been used to induce vibrations in diamagnetically levitated water droplets^27^,^28^,^38^. The air flow rate, and hence the torque on the drop, was controlled using an in-line regulator valve. After spin-up, we held the angular velocity of the droplet at a constant value for at least 10 s before solidification commenced. This was to ensure that the droplet reached a state of solid body rotation, and that the surface vibrations, excited by transient accelerations imparted during spin-up, had time to decay. After solidification, drops were kept levitating within the magnet for a further 5 minutes to ensure that the wax shape was entirely solid before removal for examination. The lengths of the three symmetry axes were measured using a digital vernier caliper.
